# Monitoring and Characterization of Oseltamivir-Resistant Pandemic (H1N1) 2009 Virus, Japan, 2009–2010

**DOI:** 10.3201/eid1703.101188

**Published:** 2011-03

**Authors:** Makoto Ujike, Miho Ejima, Akane Anraku, Kozue Shimabukuro, Masatsugu Obuchi, Noriko Kishida, Xu Hong, Emi Takashita, Seiichiro Fujisaki, Kazuyo Yamashita, Hiroshi Horikawa, Yumiko Kato, Akio Oguchi, Nobuyuki Fujita, Masato Tashiro, Takato Odagiri, the Influenza Virus Surveillance Group of Japan

**Affiliations:** Author affiliations: National Institute of Infectious Diseases, Tokyo, Japan (M. Ujike, M. Ejima, A. Anraku, K. Shimabukuro, M. Obuchi, N. Kishida, X. Hong, E. Takashita, S. Fujisaki, K. Yamashita, M. Tashiro, T. Odagiri);; National Institute of Technology and Evaluation, Tokyo (H. Horikawa, Y. Kato, A. Oguchi, N. Fujita)

**Keywords:** Influenza virus, viruses, influenza, oseltamivir, antimicrobial resistance, oseltamivir, neuraminidase, pandemic (H1N1) 2009, expedited, research

## Abstract

To monitor and characterize oseltamivir-resistant (OR) pandemic (H1N1) 2009 virus with the H275Y mutation, we analyzed 4,307 clinical specimens from Japan by neuraminidase (NA) sequencing or inhibition assay; 61 OR pandemic (H1N1) 2009 viruses were detected. NA inhibition assay and M2 sequencing indicated that OR pandemic (H1N1) 2009 virus was resistant to M2 inhibitors, but sensitive to zanamivir. Full-genome sequencing showed OR and oseltamivir-sensitive (OS) viruses had high sequence similarity, indicating that domestic OR virus was derived from OS pandemic (H1N1) 2009 virus. Hemagglutination inhibition test demonstrated that OR and OS pandemic (H1N1) 2009 viruses were antigenically similar to the A/California/7/2009 vaccine strain. Of 61 case-patients with OR viruses, 45 received oseltamivir as treatment, and 10 received it as prophylaxis, which suggests that most cases emerged sporadically from OS pandemic (H1N1) 2009, due to selective pressure. No evidence of sustained spread of OR pandemic (H1N1) 2009 was found in Japan; however, 2 suspected incidents of human-to-human transmission were reported.

## Abstract 2

No evidence of sustained spread was found, but 2 incidents of human-to-human transmission were suspected.

In March and early April of 2009, a new swine-origin A/H1N1 influenza virus, now called pandemic (H1N1) 2009, emerged in Mexico and the United States and spread rapidly (*1**–**3*). On June 11, 2009, the World Health Organization (WHO) declared a phase-6 pandemic alert, indicating a global pandemic. The earliest virus isolates were sensitive to the neuraminidase inhibitors (NAIs) zanamivir and oseltamivir, but resistant to M2 inhibitors, such as amantadine and rimantadine (*1**,**3**–**5*). Thus, the NAIs have been used globally for treatment and prophylaxis of pandemic (H1N1) 2009 virus inflection.

Oseltamivir-resistant (OR) pandemic (H1N1) 2009 was first detected in Japan, Denmark, and Hong Kong during May–June 2009 and has since been sporadically identified around the world (*6**–**8*). The OR pandemic (H1N1) 2009 viruses have a specific NA mutation, a histidine-to-tyrosine substitution at amino acid position 275 (N1 numbering, H275Y), that confers resistance to oseltamivir. In a report of 39 OR pandemic (H1N1) 2009 cases (as of October 22, 2009), 16 were associated with treatment, 13 were associated with postexposure prophylaxis, 3 were in NAI-untreated patients, and 7 were of unknown association (*8*). Preliminary global NAI surveillance showed 190 OR pandemic (H1N1) 2009 infections among >15,000 clinical specimens; thus, the global frequency of OR pandemic (H1N1) 2009 was <1.5% (as of January 8, 2010) (*9*). These reports indicated that human-to-human transmission of OR pandemic (H1N1) 2009 was limited but that oseltamivir treatment and prophylaxis could lead to emergence of OR pandemic (H1N1) 2009 virus.

A report for 1997–2007 showed that Japan accounted for ≈70% of the world’s oseltamivir consumption (*10*). From August 2009 to March 2010, 9.76 million doses of oseltamivir were supplied in Japan, ≈2.3× that of the 2008–09 seasons (data from Chugai Co. Ltd, Tokyo, Japan). Thus, Japan is a high-risk environment for the development of OR pandemic (H1N1) 2009 virus because of drug use pressure. The emergence of such resistance is alarming, because OR seasonal influenza A (H1N1) viruses can rapidly spread worldwide once they acquire the capacity for human-to-human transmission (*11**–**15*). Additionally, in the 2009–10 season in Japan, almost all cases of influenza were caused by pandemic (H1N1) 2009 viruses (Figure 1). Thus, close surveillance must be maintained to detect pandemic (H1N1) 2009 and changes in its transmissibility and genetic and antigenic characteristics.

**Figure 1:**
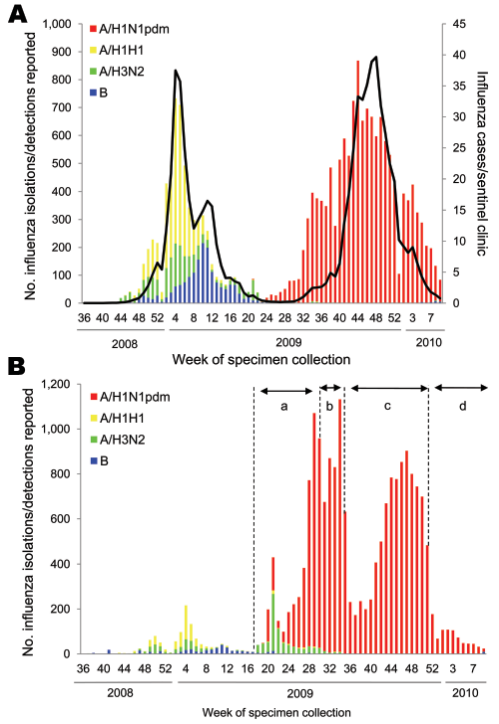
Weekly cases of influenza and isolation or detection of influenza viruses by influenza sentinel clinics (A) and nonsentinel clinics (B) from week 36 of 2008 to week 9 of 2010 in Japan (as of March 9, 2010). Pandemic (H1N1) 2009 (A/H1N1pdm) surveillance in Japan was divided into 4 stages depending on the prevalence situation, as shown in panel B: a) case-based surveillance (April 28–July 23), b) outbreak and hospitalization surveillance (July 24–August 24), c) hospitalization surveillance (August 25–December 20), and d) severe/fatal case surveillance (December 21 onwards). The sentinel clinics, consisting of 3,000 pediatric clinics and 2,000 internal medical clinics, collected samples randomly, while the nonsentinel clinics collected samples depending on the surveillance stage. Local public health laboratories randomly selected these samples for neuraminidase (NA) surveillance from both sentinel and nonsentinel clinics. In this study, 4,307 clinical specimens, comprising both original samples (n = 440) and isolates (n = 3,867), were subjected to full or partial NA sequencing for detection of the H275Y mutation. All oseltamivir-resistant (n = 61) and ≈10% of oseltamivir-susceptible pandemic (H1N1) 2009 (n = 421) isolates were then subjected to NA assay. The treatment history of the 4,307 cases consists of NA inhibitor–untreated (n = 1,088), oseltamivir use (n = 516), zanamivir use (n = 103), and unknown history (n = 2,600). Black line in panel A indicates weekly cases of influenza-like illness per influenza sentinel clinic.

We monitored and characterized 4,307 clinical specimens collected in Japan during May 2009–February 2010 from patients with OR pandemic (H1N1) 2009 by NA sequencing, NAI assay, or both. Of them, we found 61 OR pandemic (H1N1) 2009 viruses with the H275Y mutation.

## Materials and Methods

### Virus Testing

Influenza sentinel clinics and nonsentinel institutes send original samples to local public health laboratories for detection and virus isolation. In total, 4,307 clinical specimens, comprising both original samples (n = 440) and clinical isolates (n = 3,867), underwent either full or partial (nt 695–1110) NA sequencing to detect the H275Y mutation. Samples from 1,088 cases were collected before oseltamivir exposure, 516 were associated with oseltamivir use, 103 were associated with zanamivir use, and for 2,600, antivirual treatment status was unknown. We collected all OR pandemic (H1N1) 2009 isolates and randomly selected OS isolates (≈10%) from local public health laboratories. These representative OS and OR pandemic (H1N1) 2009 isolates underwent NA inhibition assay (421 OS and 61 OR viruses tested), full NA and hemagglutination (HA) sequencing (190 OS and 61 OR), internal gene (PB2/PB1/PA/NP/M/NS) sequencing (138 OS and 20 OR), and hemagglutination inhibition (HI) test (583 OS and 59 OR).

### Sequence Analysis

Phylogenetic trees of NA and HA genes were constructed by neighbor-joining method. A phylogenetic tree was constructed by using representative OR and OS pandemic (H1N1) 2009 isolates from several prefectures of Japan. Sequence information of pandemic (H1N1) 2009 from other countries was downloaded from the Global Initiative on Sharing Avian Influenza Data (GISAID) and GenBank. All amino acid positions in the phylogenetic tree were described by N1 numbering.

### NAI Assay

A chemiluminescent NAI assay was performed with the NA-star kit (Applied Biosystems, Tokyo, Japan) (*13*). Briefly, final drug concentration was 0.03–6,500 nM for oseltamivir and 0.03–12,500 nM for zanamivir. Chemiluminescence was assayed with an LB940 plate reader (Berthhold Technologies, Bad Wilbad, Germany). Drug concentrations required for 50% inhibitory concentration of NA activity (IC_50_) were calculated with MikroWin 2000 software (ver. 4; Mikrotek Laborsysteme GmbH, Overath, Germany). To validate the NAI assay, we used already characterized drug-resistant viruses and sensitive counterparts as controls: A/Hokkaido/15/2002 (155H) and A/Hokkaido/9/2002 (155Y), zanamivir (*16*); A/Denmark/528/2009pdm (275Y), A/Denmark/524/2009pdm (275H), seasonal-H1N1 A/Yamagata/68/2008 (275Y), A/Yamagata/41/2008 (275H), oseltamivir.

### Statistical Analyses

Box-and-whisker plots were used to determine the cutoff value between NAI-resistant (outlier) and -sensitive viruses. The box contains 50% of the results, representing the middle 2 quartiles (25%–75%). The length of the box shows the interquartile range (IQR). The cutoff value was defined as the upper quartile + 3.0 × interquartile range from the 25th to 75th percentile. For statistical analyses, OR pandemic (H1N1) 2009 viruses with the H275Y mutation were excluded from the overall population.

### HI Test

An HI test was performed to evaluate the reactivity of ferret antiserum against the 2009/10 vaccine strain A/California/7/2009, as described in the WHO Manual (*17*). The efficacy of ferret postinfection antiserum against egg-grown A/California/7/2009 was used as a reference. Antiserum was treated with receptor-destroying enzyme II (Denka Seiken, Tokyo, Japan) and adsorbed with turkey erythrocytes before testing, to prevent nonspecific reactions. A 0.5% suspension of turkey erythrocytes was used for the HI test.

## Results

### Geographic Distribution of OR Pandemic (H1N1) 2009

**Figure 2:**
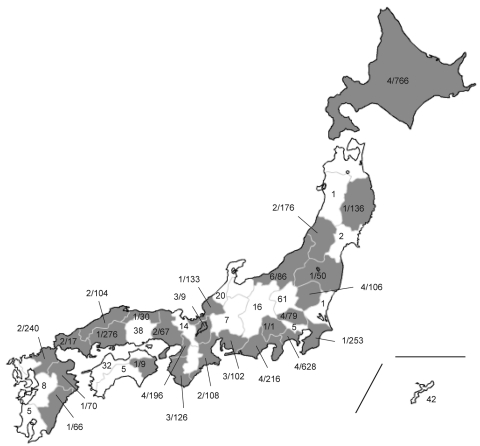
Geographic distribution of H275Y-harboring oseltamivir-resistant pandemic (H1N1) 2009 viruses in Japan, May 2009–February 2010. Values are no. oseltamivir-resistant isolates/total no. tested. Overall prevalence in Japan was 1.4% (61/4,307).

**Figure 3:**
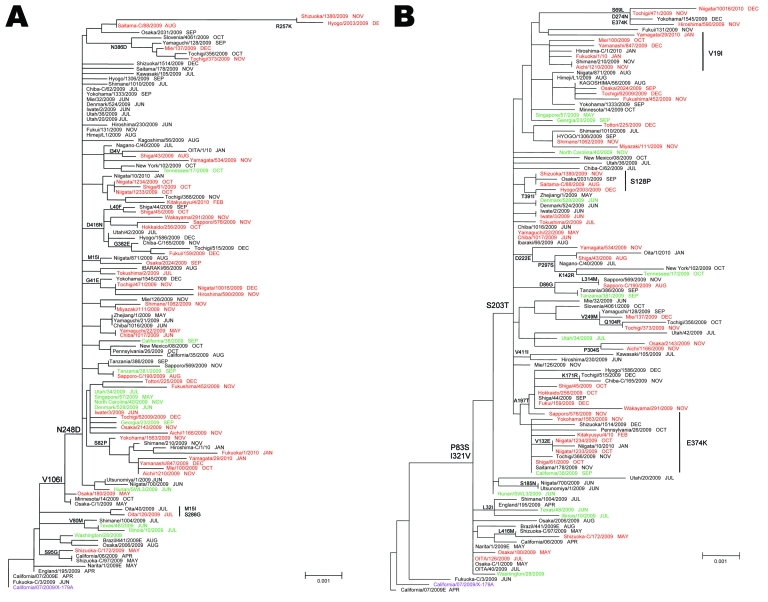
Phylogenetic analysis of influenza pandemic (H1N1) 2009 viruses neuraminidase (NA) (A) and hemagglutinin (HA) genes (B). Most pandemic (H1N1) 2009 viruses possessed the amino acid substitutions S203T in HA and V106I and N248D in NA. Red, oseltamivir-resistant pandemic (H1N1) 2009 from Japan; green, oseltamivir-resistant pandemic (H1N1) 2009 from outside Japan; black, oseltamivir-susceptible (OS) pandemic (H1N1) 2009; purple, 2009–10 current vaccine strains. The sampling month of each isolate is listed following the strain name. The phylogenetic tree of NA and HA genes was constructed by using the neighbor-joining method. Scale bars indicate nucleotide substitutions per site.

The 4,307 clinical specimens isolated during May 2009–February 2010 were collected from 41 of 47 prefectures in Japan, and the H275Y mutation was detected by NA sequencing. In total, 61 (1.4%) OR pandemic (H1N1) 2009 viruses possessed the H275Y (n = 48) or 275H/Y mixed (n = 13) mutations (Figure 2). OR pandemic (H1N1) 2009 emerged sporadically in several prefectures and was detected over a period of several months (Figure 2, Figure 3).

### Patient Treatment History and Epidemiologic Background

Of 4,307 case-patients, 516 had oseltamivir treatment, 103 had zanamivir treatment, 1,088 were NAI-untreated, and the treatment history of 2,600 was unknown. Of the 61 cases of OR pandemic (H1N1) 2009, 45 were associated with oseltamivir treatment, 10 with postexposure prophylaxis, and 6 occurred in NAI-untreated patients; thus, oseltamivir treatment and prophylaxis likely accelerated emergence of OR pandemic (H1N1) 2009. The relationship between time of sampling (days after oseltamivir treatment) and OR pandemic (H1N1) 2009 detection showed that OR pandemic (H1N1) 2009 was generally detected at least 4 days after oseltamivir treatment (Table 1).

**Table1:** Relationship between detection of oseltamivir-resistant pandemic (H1N1) 2009 and interval from oseltamivir treatment to sample collection, Japan, 2009–2010*

No. samples	Days after oseltamivir treatment, N = 516†											
	Unknown	0‡	1	2	3	4	5	6	7	8	9	>10
Total no.	169	54	116	54	37	31	36	7	6	1	3	2
No. oseltamivir-resistant pandemic (H1N1) 2009	3	0	4	2	4 (1)	13 (1)	19 (1)	3 (2)	2	1 (1)	2 (2)	2 (2)

*Of total 4,307 specimens tested, neuraminidase inhibitor treatment history was available for 1,707; of these specimens, 516 were from patients who had received oseltamivir treatment. †Parentheses indicate prophylactic use, e.g., 4 (1) = 1 of 4 total uses was for prophylaxis. ‡Day 0 represents the samples collected within 24 h after oseltamivir use.

Of the 61 case-patients, 36 (59%) were male; 19 (31%) were 0–4 years, 25 (41%) were 5–18 years, 12 (20%) were 19–50 years, and 5 (8%) were >50 years. Underlying medical conditions were known for 41; 24 had chronic underlying medical conditions (pulmonary [13], neurologic [4], blood [3], diabetes [1], kidney disease [1], immunocompromised [5], other conditions [2]).

### Two Possible Cases of Human-to-Human Virus Transmission

Almost all OR pandemic (H1N1) 2009 cases emerged sporadically and were not epidemiologically linked. However, 2 cases of human-to-human transmission were suspected. One was observed in Niigata Prefecture where 2 children hospitalized in the same room were infected with OR pandemic (H1N1) 2009 virus within a few days. Symptoms developed first in a 4-year-old girl on October 10, 2009, and she received oseltamivir. OR pandemic (H1N1) 2009 virus was isolated from this patient on October 14. A 6-year-old boy in the same room received prophylaxis (by treatment dosage) with oseltamivir beginning October 10. However, he experienced symptoms on October 13, and OR pandemic (H1N1) 2009 virus was isolated on October 14. The patients were in a double room, and patients with pandemic (H1N1) 2009 were not around them. Genetic analyses of the 2 viruses (i.e., A/Niigata/1233/2009 and A/Niigata/1234/2009) showed only 1 aa difference (D to G), at position 256 in PB2, and they also shared unique changes in NS1 and in PB1 (Table 2). Transmission possibilities were as follows: 1) OR pandemic (H1N1) 2009 was directly transmitted from the female patient or 2) an OS pandemic (H1N1) 2009 was transmitted from the female patient and an OR pandemic (H1N1) 2009 virus emerged in the male patient.

**Table 2:** Amino acid differences of each internal protein between oseltamivir-resistant (n = 20) and oseltamivir-sensitive (n = 138) pandemic (H1N1) 2009 virus, by strain, Japan, 2009–2010*

Strain	M1	M2	NP	NS1	NS2	PA	PB1†	PB2†
A/Niigata/1459/2009						V122I	**I435V**, N537S	
A/Osaka/2024/2009							**I435V**	R251K
A/Shimane/1062/2009						V127A, T357I	**I435V**	N448S
A/Shimane/188/2009				A102T			**I435V**	R54K
A/Yokohama/1340/2009						S186N	**I435V**	
A/Yokohama/1394/2009							**I435V**, F466Y	
A/Shiga/61/2009			V119I	M93I			**I435V**, A93V, T257A	**K660R**
A/Niigata/1233/2009				M93I, E217K			T257A	**K660R**
A/Niigata/1234/2009				M93I, E217K			T257A	**K660R**, D256G
A/Shiga/45/2009								**K660R**
A/Chiba/1017/2009								
A/Iwate/3/2009	K103R‡							
A/Mie/100/2009		S23N		T94N			V609A	R251K
A/Oita/126/2009			I100V	E55G, V103I		L370I	K480R	
A/Osaka/180/2009	A33T			V103I	E63K		I667T	V649I, E700K
A/Saitama-C/88/2009				E208K	M50I	A70V		V227I
A/Sapporo-C/190/2009		D21G						
A/Shiga/43/2009							A652T	
A/Tokushima/2/2009						M311I		
A/Yamaguchi/22/2009						V379I		

*M, matrix protein; NP, nucleoprotein; NS, nonstructural protein; PA, polymerase A; PB, polymerase B. †Of the 138 oseltamivir-sensitive pandemic (H1N1) 2009 virus samples, I435V and K660R (**boldface**) were observed from 32 and 12 isolates, respectively. These changes would sporadically occur in both pandemic (H1N1) 2009 isolate types. ‡Lys (K) at position 103 in M1 protein, consensus amino acid among the oseltamivir-resistant pandemic (H1N1) 2009 virus, was replaced with Arg (R).

The other suspected instance of human-to-human transmission occurred in Tottori Prefecture. In a 9-year-old boy, symptoms developed on December 18, 2009, and OR pandemic (H1N1) 2009 virus was isolated from a sample collected on the same day, before oseltamivir use. However, the patient’s 2 brothers were both infected with pandemic (H1N1) 2009 virus and had received oseltamivir since December 15. Although samples from these persons were not available, OR pandemic (H1N1) 2009 likely emerged in 1 patient and was transmitted to the other.

### Case Unrelated to Oseltamivir Use

Detailed epidemiologic information was available for 2 of 6 persons with OR pandemic (H1N1) 2009 infections untreated by NAIs. Besides the case in Tottori Prefecture, another occurred in Oita Prefecture. The index patient had a mild cough beginning on July 12, and typical influenza symptoms developed on July 15. OR pandemic (H1N1) 2009 virus was detected in a sample taken on July 16, before oseltamivir use. However, symptoms had developed in the index patient’s son on July 11; the boy received zanamivir on July 12 (OR pandemic (H1N1) 2009 virus was not detected from a sample taken that day). No reports have indicated that zanamivir can induce OR virus with the H275Y mutation. The OR pandemic (H1N1) 2009 virus may have thus emerged naturally, with no selective pressure. However, the index patient may have been exposed to an oseltamivir-treated person outside of her household who harbored OR pandemic (H1N1) 2009 virus.

### Genetic Analysis

Phylogenetic analyses of the HA and NA genes showed that most shared amino acid changes: S203T in HA and V106I and N248D in NA (Figure 3). In both trees, OR pandemic (H1N1) 2009 isolates were genetically scattered and possessed several sporadic amino acid changes, but each OR pandemic (H1N1) 2009 was genetically close to OS pandemic (H1N1) 2009 (Figure 3). Several OR pandemic (H1N1) 2009 isolates from Japan were also closely related to OR pandemic (H1N1) 2009 isolates from other countries.

Analysis of the genomes of representative OR (n = 20) and OS pandemic (H1N1) 2009 (n = 138) provided further insight into their similarities. First, comparison of the internal amino acid sequences of each OR pandemic (H1N1) 2009 and OS pandemic (H1N1) 2009 isolate consensus showed that OR viruses possessed several sporadic amino acid changes, but did not exhibit any common amino acid changes unique to OR pandemic (H1N1) 2009 viruses, indicating that the internal genes of OR and OS pandemic (H1N1) 2009 viruses were genetically indistinguishable (Table 2). Second, comparison of a 2 samples from a patient with pandemic (H1N1) 2009 before and after oseltamivir treatment (A/Chiba/1016/2009 and A/Chiba/1017/2009) showed only the H275Y change in NA and no changes in any other proteins. Finally, no evidence of reassortment of pandemic (H1N1) 2009 and seasonal influenza A (H1N1) viruses was detected.

**Figure A1:**
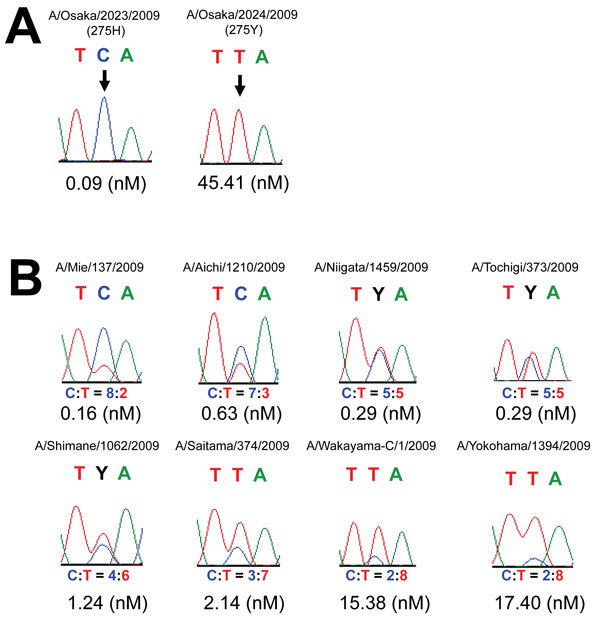
The relationship between sequence wave patterns and 50% inhibitory concentration (IC_50_) values of representative oseltamivir-susceptible (OS) and oseltamivir-resistant (OR) pandemic (H1N1) 2009 viruses and mixed viruses. A) Neuraminidase (NA) sequences and IC_50_ values of OS- (A/OSAKA/2023/2009, 275H) and OR pandemic (H1N1) 2009 (A/OSAKA/2024/2009, 275Y). The codon TCA (amino acid H at position 275 in NA protein) of OS pandemic (H1N1) 2009 changed to TTA (Y) in OS pandemic (H1N1) 2009 (arrow). Almost all viruses tested showed a single wave pattern at this position. IC_50_ values and the C:T signal ratio are indicated below each sequence. B) NA sequences and IC_50_ values of representative mixed viruses. Several wave patterns were observed among mixed viruses. The IC_50_ value of mixed viruses that had a C:T signal ratio of 8:2–5:5 tended to be close to that of OS pandemic (H1N1) 2009 (range 0.16–0.63 nM), while the IC_50_ values of mixed viruses that had a C:T signal ratio of 2:8 tended to be close to that of OS pandemic (H1N1) 2009 (15.– 17.40 nM). The IC_50_ values of mixed viruses that had a C:T signal ratio of 4:6–3:7 tended to display middling IC_50_ values (range 1.24–2.14 nM). In most cases, the C:T signal ratios of mixed viruses were correlated with IC_50_ values.

Of 61 pandemic (H1N1) 2009 OR isolates, those from 13 patients were of mixed NA gene populations (H275 and Y275). Because all 13 patients had received oseltamivir, these samples would have been collected during selective pressure–induced generation of OR pandemic (H1N1) 2009 from OS pandemic (H1N1) 2009 (Figure A1). Because calculating precise IC_50_ values from a mixed population of NAI-resistant and -sensitive viruses is not possible (*13**,**18*), the 13 mixed isolates were excluded from the overall population for the purposes of the statistical analysis of OR.

### Antiviral Drug Susceptibility

NAI data are summarized in Table 3. The average IC_50_ value of OR pandemic (H1N1) 2009 (n = 48) for oseltamivir was 370-fold higher than that of OS pandemic (H1N1) 2009 (n = 421) viruses. For zanamivir, 3 of 482 viruses were identified as outliers (cutoff >0.60 nM). Compared with the consensus sequence of OS pandemic (H1N1) 2009, one OS pandemic (H1N1) 2009 A/Okayama/17/2009pdm (0.61 nM) had a D151D/N mixture in its NA protein, and 2 OR pandemic (H1N1) 2009, A/Shiga/43/2009pdm (0.64 nM) and A/Yokohama/1538/2009pdm (0.64 nM) possessed I34V and I195V substitutions in the NA protein, respectively (Table A1). The IC_50_ values of OS and OR pandemic (H1N1) 2009 viruses were similar to those of their seasonal influenza A (H1N1) counterpart viruses (Table 3).

**Table 3:** Summary of neuraminidase inhibition assay of oseltamivir-resistant and oseltamivir-sensitive pandemic (H1N1) 2009 virus to oseltamivir and zanamivir*

Strain	IC_50_, (nM/L)						
	Oseltamivir				Zanamivir		
	No. isolates	Mean ± SD (range)	Cutoff value		No. isolates	Mean ± SD (range)	Cutoff value
Pandemic (H1N1) 2009							
Oseltamivir-sensitive	421	0.10 ± 0.02 (0.05–0.19)	>0.20		421	0.28 ± 0.06 (0.11–0.61)	>0.60†
Oseltamivir-resistant	48‡	37.28 ± 14.06 (20.69–80.91)	NC		61	0.36 ± 0.11 (0.17–0.64)	
Seasonal influenza (H1N1) (A/Yamagata/41/2008)							
Oseltamivir-sensitive		0.09 ± 0.02§				0.24 ± 0.10	
Oseltamivir-resistant		51.76 ± 9.54				0.37 ± 0.13	

*IC_50_, 50% inhibitory concentration; NC, not calculated. †Because both IC_50_ values of OS and OR pandemic (H1N1) 2009 viruses were indistinguishable, the cutoff values for zanamivir were calculated from the overall population (N = 482). ‡IC_50_ values of 13 mixed samples with H275 and Y275 were excluded from overall population in statistical analysis of OR isolates. §Mean ± SD IC_50_ values of control seasonal influenza A (H1N1) viruses were determined from 10 independent experiments for oseltamivir and 2 for zanamivir.

Susceptibility to M2 inhibitors was determined by M2 sequencing. All tested viruses, including OR (n = 20) and OS pandemic (H1N1) 2009 (n = 138), had an S31N resistance marker in the M2 protein, suggesting that all pandemic (H1N1) 2009 isolates were resistant to M2 inhibitors.

### Antigenic Characterization

The HI test was performed to estimate the reactivity of OS (n = 583) and OR pandemic (H1N1) 2009 (n = 59) virus to ferret antiserum against the 2009–10 vaccine strain A/California/7/2009. More than 93% of OS (n = 546) and OR pandemic (H1N1) 2009 (n = 55) isolates were inhibited by anti-A/California/7/2009 ferret antiserum, and 5.8% and 5.1% of OS (n = 34) and OR pandemic (H1N1) 2009 (n = 3), respectively, showed a 4-fold reduced HI titer. Only 0.5% and 1.7% of OS- (n = 3) and OR pandemic (H1N1) 2009 (n = 1), which had either the K153E or G155E changes in deduced antigenic sites in HA protein, showed at least an 8-fold reduction in HI titer. Thus, OS and OR pandemic (H1N1) 2009 are antigenically indistinguishable and similar to the 2009–10 current vaccine strain A/California/7/2009.

## Discussion

The data presented here provide no evidence of sustained spread of OR pandemic (H1N1) 2009 in Japan. In this study, clinical specimens were collected from both NAI-untreated and NAI-treated patients, so later samples were collected after the exertion of selective pressure by drug treatment. However, frequency of detection of OR pandemic (H1N1) 2009 was low (1.4%). Because OR and OS pandemic (H1N1) 2009 isolates were genetically and antigenically indistinguishable, the current 2009–10 vaccine would be expected to be effective against recent OR pandemic (H1N1) 2009. No evidence of reassortment with seasonal influenza A (H1N1) virus was detected. Immunocompetent patients infected with OR pandemic (H1N1) 2009 showed typical uncomplicated influenza symptoms, similar to those caused by OS pandemic (H1N1) 2009 (*19*).

Early reports suggested that ≈70% of the worldwide consumption of oseltamivir occurs in Japan (*10*), but long-term NAI surveillance in Japan from 1996 to 2007 (*10*) and previous NAI surveillance (*16**,**20**,**21*) showed a low frequency of resistant viruses, suggesting that the transmissibility of OR viruses selected by drug pressure was remarkably reduced. However, beginning in November 2007, an unexpectedly high frequency of OR seasonal influenza A (H1N1) viruses with the H275Y mutation was detected in Europe (*11**–**15*). Most were isolated from NAI-untreated patients and were more transmissible than OS influenza A (H1N1), resulting in rapid global dissemination (*15*). In contrast, even in the 2007–08 season, OR influenza A (H1N1) was detected only rarely (1.5%–2.6%) in Japan, despite the high level of oseltamivir use (*13**,**22*). However, OR influenza A (H1N1) virus was detected at a far higher frequency (≈100%) the next year (*13*). Thus, the pattern of oseltamivir use did not correspond to the emergence and widespread distribution of OR influenza A (H1N1) viruses.

In contrast, this study and a recent report (*8*) found that OR pandemic (H1N1) 2009 has been detected predominantly in isolates from oseltamivir recipients. Unlike recent OR influenza A (H1N1) viruses, such OR pandemic (H1N1) 2009 viruses seemed to have restricted transmissibility among humans. These findings indicated that oseltamivir use was responsible for the emergence of OR pandemic (H1N1) 2009 viruses, but perhaps not for the widespread distribution of OR pandemic (H1N1) 2009.

Although the reason why recent OR seasonal influenza A (H1N1) isolates did not lose fitness remains unclear, a functional defect in NA proteins caused by H275Y may be counteracted by permissive secondary mutations. Two such mutations, R222Q and V234M, have been identified in seasonal influenza (H1N1) (*23*). Although whether the amino acids of the corresponding positions of pandemic (H1N1) 2009 play a similar role is unknown, the NA protein of this virus does have A and V residues at positions 222 and 234, respectively; a V at position 234 was identical to that in a nonpermissive amino acid sequence. Other sporadic and some shared amino acid change(s) were observed in the NA protein of OR pandemic (H1N1) 2009 viruses, but these changes apparently did not restore viral fitness, because no efficiently transmissible OR pandemic (H1N1) virus was found. These observations suggest that the NA proteins of recent OR pandemic (H1N1) 2009 isolates likely did not possess such permissive secondary mutation(s) (Table A1).

However, all recent animal studies of OR pandemic (H1N1) 2009 virus have shown that viral fitness and transmissibility did not differ from those of OS pandemic (H1N1) 2009 virus (*24**–**26*) and had a potential to supersede OS pandemic (H1N1) 2009 virus. Nevertheless, OR pandemic (H1N1) 2009 did not supersede OS pandemic (H1N1) 2009 in humans. This inconsistency may be explained by differences in infectious dose used in the animal models.

In an NAI assay of zanamivir susceptibility, statistical analysis identified 3 outliers. One OS pandemic (H1N1) 2009 possessed the D151D/N mutation, which has been reported to affect susceptibility to zanamivir in seasonal viruses (*20**,**21*). Two OR pandemic (H1N1) 2009 isolates had the substitutions I34V and I195V in NA; however, whether these affect zanamivir susceptibility is unclear. We also assayed peramivir susceptibility in representative OR and OS pandemic (H1N1) 2009 isolates. Data suggested that OR pandemic (H1N1) 2009 virus, which contained the H275Y substitution, possessed cross-resistance to peramivir, as reported by another group (*27*).

Both case reports and preliminary NAI surveillance (*8**,**19**,**28**–**32*) have indicated 2 groups are at high risk for the generation of resistant viruses. The first is patients with severely compromised or suppressed immune systems, who shed virus for prolonged periods and thus have an increased chance of developing resistant virus (*33**,**34*). WHO reported that 25% of 285 resistant cases (as of April 17, 2010) occurred in immunocompromised patients (*35*). The second group is persons who are receiving postexposure prophylaxis, who take a subtherapeutic dose of 75 mg 1×/day (treatment dosage is 75 mg 2×/day). This regimen may only partially inhibit viral replication, thus facilitating the emergence of OR pandemic (H1N1) 2009. WHO recommends chemoprophylaxis only for persons who have a higher risk for severe or complicated illness (*19*). These groups were observed in our study.

We found that most OR pandemic (H1N1) 2009 virus was detected in samples collected at least 4 days after oseltamivir treatment or prophylaxis (Table 1). However, the frequency of OR pandemic (H1N1) 2009 in each day of treatment with oseltamivir could not be calculated because of a lack of treatment history data. This timing is consistent with that for OR seasonal influenza A (H1N1, H3N2) and pandemic (H1N1) 2009 viruses, which typically emerge 3–6 days after oseltamivir treatment (*36**–**38*). Additionally, we also observed the rapid emergence of OR pandemic (H1N1) 2009 virus within 48 hours of oseltamivir exposure (*39*). Nontheless, 6 cases occurred in untreated patients. Indeed, the OR pandemic (H1N1) 2009 in the Oita case may likely be a natural occurrence. We are aware of only 2 other reports, one from Vietnam and one from Hong Kong, of naturally occurring OR pandemic (H1N1) 2009 in untreated patients (*7**,**40*).

The greatest concern regarding OR pandemic (H1N1) 2009 is that drug-resistant viruses will acquire the ability to be transmitted efficiently among humans as has recent OR seasonal influenza A (H1N1). Two hospital outbreaks in the United Kingdom and the United States have been reported (*31**,**32*). In both, the immune systems of all patients were severely compromised or suppressed, indicating that these patients had an increased risk for not only the emergence of OR pandemic (H1N1) 2009 virus, but also OR seasonal influenza (H1N1) virus (*31**,**32*). In contrast, particular attention should be paid to the Vietnamese case because a naturally occurring OR pandemic (H1N1) 2009 virus caused a cluster of 7 cases in immunocompetent patients with no history of oseltamivir use (*40*).

Despite the high level of oseltamivir use in Japan, prevalence of OR pandemic (H1N1) 2009 remains low (1.4%). Thus, oseltamivir remains the first option for treating pandemic (H1N1) 2009, but zanamivir should be considered for immunocompromised patients. Additionally, as first priority for prophylaxis of both OR and OS pandemic (H1N1) 2009 infection should be vaccination, but not antiviral agents. Conversely, a preclinical animal model showed that OR pandemic (H1N1) 2009 had high potential to acquire transmissibility without losing viral fitness (*24**–**26*). Whether and how OR pandemic (H1N1) 2009 may acquire efficient transmissibility among humans are not known. Thus, vigilant monitoring of OR pandemic (H1N1) 2009 infection and alterations in its transmissibility and antigenic and genetic characteristics is essential.

Members of the Influenza Virus Surveillance Group of Japan: Hideki Nagano (Hokkaido Institute of Public Health), Masayuki Kikuchi (Sapporo City Institute of Public Health), Masaki Takahashi (Research Institute for Environmental Sciences and Public Health of Iwate Prefecture), Yuki Sato (Miyagi Prefectural Institute of Public Health and Environment), Masanori Katsumi (Sendai City Institute of Public Health), Hiroyuki Saito (Akita Research Center for Public Health and Environment), Katsumi Mizuta (Yamagata Prefectural Institute of Public Health), Syoko Hirose (Fukushima Prefectural Institute of Public Health), Setsuko Fukaya (Ibaraki Prefectural Institute of Public Health), Teruko Oogane (Tochigi Prefectural Institute of Public Health and Environmental Sciences), Hiroyuki Tsukagoshi (Gunma Prefectural Institute of Public Health and Environmental Sciences), Yuka Uno (Saitama City Institute of Health Science and Research), Hiromi Maru (Chiba Prefectural Institute of Public Health), Hajime Yokoi (Chiba City Institute of Health and Environment), Mami Nagashima (Tokyo Metropolitan Institute of Public Health), Sumi Watanabe (Kanagawa Prefectural Institute of Public Health), Hideaki Shimizu (Kawasaki City Institute of Public Health), Sumiko Ueda (Sagamihara City Laboratory of Public Health), Miyako Kon (Niigata Prefectural Institute of Public Health and Environmental Sciences), Gen Kobayashi and Yoko Miyajima (Niigata City Institute of Public Health and Environment), Sanae Kuramoto (Ishikawa Prefectural Institute of Public Health and Environmental science), Masako Nakamura (Fukui Prefectural Institute of Public Health), Hiroyoshi Asakawa (Yamanashi Institute for Public Health), Seiko Sawatari (Gifu Prefectural Institute of Health and Enviromental Sciences), Yasunori Tanaka (Gifu Municipal Institute of Public Health), Toshihiro Yamada (Shizuoka Institute of Environment and Hygiene), Shinobu Ide (Shizuoka City Institute of Environmental Sciences and Public Health), Yoshinori Kohno (Hamamatsu City Health Environment Research Center), Yoshihiro Yasui (Aichi Prefectural Institute of Public Health), Noriko Goto (Nagoya City Public Health Research Institute), Takuya Yano (Mie Prefecture Health and Environment Research Institute), Fumie Matsumoto (Shiga Prefectural Institute of Public Health), Tohru Ishizaki (Kyoto Prefectural Institute of Public Health and Environment), Satoshi Hiroi (Osaka Prefectural Institute of Public Health), Hideyuki Kubo (Osaka City Institute of Public Health and Environmental Sciences), Kiyoko Uchino (Sakai City Institute of Public Health), Tomohiro Oshibe (Hyogo Prefectural Institute of Public Health and Consumer Sciences), Ai Mori (Kobe Institute of Health), Yoshiteru Kitahori (Nara Prefectural Institute for Hygiene and Environment), Fumio Terasoma (Wakayama Prefectural Research Center of Environment and Public Health), Takayuki Hirooka and Hidenobu Egawa (Wakayama City Institute of Public Health), Yoshiaki Kimura (Tottori Prefectural Institute of Public Health and Environmental Science), Tamaki Omura (Shimane Prefectural Institute of Public Health and Environmental Science), Shinichi Takao (Center for Public Health and Environment, Hiroshima Prefectural Technology Research Institute), Katsuhiko Abe (Hiroshima City Institute of Public Health), Yumiko Kawakami (Tokushima Prefectural Centre for Public Health and Environmental Sciences), Satomi Aoki (Ehime Prefecture Institute of Public Health and Environmental Science), Daisuke Kawamoto (Fukuoka City Institute for Hygiene and the Environment), Takayuki Hirano (Saga Prefectural Institute of Public Health and Pharmaceutical Research), Manabu Hirano (Nagasaki Prefectural Institute for Environment Research and Public Health), Seiya Harada (Kumamoto Prefectural Institute of Public-Health and Environmental Science), Miki Kato (Oita Prefectural Institute of Health and Environment), Miho Miura (Miyazaki Prefectural Institute for Public Health and Environment), Kanji Ishitani (Kagoshima Prefectural Institute for Environmental Research and Pubulic Health), Katsuya Taira (Okinawa Prefectural Institute of Health and Environment), Mitsutaka Kuzuya (Okayama Prefectural Institute for Environmental Science and Public Health), Shoichi Toda (Yamaguchi Prefectural Institute of Public Health and Environment), Chiharu Kawakami (Yokohama City Institute of Health), Mayumi Konno (Kyoto City Institute of Health and Environmental Sciences), Hiroya Komoda (Kagawa Prefectural Research Institute for Environmental Sciences and Public Health), Tae Taniwaki (Kochi Public Health and Sanitation Institute), Toshitaka Minegishi (Saitama Institute of Pubulic Health), Rika Tsutsui (Aomori Prefectural Institute of Public Health and Environment), Shizuko Kasuo (Nagano Environmental Conservation Research Institute), Yuichiro Okamura (Nagano City Health Center), Eiji Horimoto (Toyama Institute of Health), Nobuyuki Sera (Fukuoka Institute of Health and Environmental Sciences), Kotaro Murase (Kitakyushu City Institute of Environmental Sciences).

## Appendix

**Table A1:** Amino acid difference(s) of OR (n = 61) from OS (n = 190) pandemic (H1N1) 2009 virus consensus sequences in neuraminidase protein*

Strains	Amino acid difference(s) of neuraminidase protein																														
	15	16	34	40	41	43	80	82	83	85	86	95	98	106	138	154	166	195	222†	234	248	257	269	275	286	289	382	386	416	430	452
	M	T	I	L	G	Q	V	S	V	L	A	S	A	I	A	P	V	I	A	V	D	R	M	Y	S	T	G	N	D	R	T
A/Niigata/1459/2009					E‡																			Y/H§							
A/Hiroshima/590/2009					E																			Y							
A/Shizuoka-C/270/2009					E																			Y							
A/Tochigi/471/2009					E																			Y							
A/Yokohama/1340/2009					E																			Y							
A/Niigata/10016/2009					E							R												Y							
A/Aichi/1210/2009								P																Y/H							
A/Kobe/92495/2009								P																Y							
A/Mie/100/2009								P																Y							
A/Saitama/374/2009								P																Y/H							
A/Wakayama/318/2009								P																Y/H							
A/Yamaguchi/248/2009								P																Y							
A/Yamanashi/847/2009								P																Y							
A/Yokohama/1563/2009								P																Y							
A/Fukuoka/1/2010							M	P																Y							
A/Yamagata/29/2010								P					T								N			Y							
A/Wakayama-C/1/2010								P							T									Y/H							
A/Niigata/10019/2009								P												A				Y							
A/Osaka/180/2009																					N			Y							
A/Oita/126/2009	I													V							N			Y	G						
A/Shizuoka-C/172/2009												G		V							N			Y							
A/Osaka/2191/2009																							I	Y							
A/Tochigi/609/2009																							I	Y/H							
A/Tochigi/612/2009																							I	Y							
A/Hokkaido/256/2009																								Y/H					N		
A/Shizuoka-C/247/2009																								Y					N		
A/Wakayama/291/2009																								Y					N		
A/Shiga/45/2009				F																				Y					N		
A/Sapporo/576/2009																S							V	Y					N		
A/Yokohama/1538/2009																		V						Y/H					N		
A/FUKUI/159/2009																								Y			E		N		
A/Osaka/2024/2009		I																						Y							
A/Shiga/43/2009			V																					Y/H							
A/Aichi/1166/2009						K																		Y							
A/Sapporo/31/2010									M															Y							
A/Shimane/1062/2009										F														Y/H							
A/Saitama/396/2009											T													Y							
A/Shiga/61/2009																	I							Y							
A/Shizuoka/1380/2009																						K		Y							
A/Hyogo/2003/2009																						K		Y							I
A/Osaka/2143/2009																								Y		I					
A/Mie/137/2009																								Y/H				D			
A/Tochigi/373/2009																								Y/H				D			
A/Aichi/1019/2009																								Y						Q	
A/Chiba/1017/2009																								Y							
A/Fukushima/452/2009																								Y							
A/Iwate/3/2009																								Y							
A/Kitakyusyu/4/2010																								Y							
A/Miyazaki/111/2009																								Y							
A/Niigata/1233/2009																								Y							
A/Niigata/1234/2009																								Y							
A/Niigata-C/186/2009																								Y							
A/Saitama/408/2009																								Y							
A/Saitama-C/88/2009																								Y							
A/Sapporo/190/2009																								Y							
A/Shimane/188/2009																								Y							
A/Tokushima/2/2009																								Y							
A/Tottori/225/2009																								Y							
A/Yamagata/534/2009																								Y							
A/Yamaguchi/22/2009																								Y							
A/Yokohama/1394/2009																								Y/H							
*OR, oseltamivir resistant; OS, oseltamivir susceptible. †Recent OR seasonal influenza A (H1N1) isolates have R222Q and V234M mutations that would restore viral fitness (*23*). ‡Of 190 pandemic (H1N1) 2009 virus isolates, G41E, S82P, D248N, M269I, and D416N were observed from 4, 10, 27, 0, and 17 OS pandemic (H1N1) 2009 virus isolates, respectively. §Mixed population of H275 and Y275.																															
